# Bio-Based Thermoplastic Starch Composites Reinforced by Dialdehyde Lignocellulose

**DOI:** 10.3390/molecules25143236

**Published:** 2020-07-16

**Authors:** Peng Yin, Wen Zhou, Xin Zhang, Bin Guo, Panxin Li

**Affiliations:** 1 Department of Chemistry, College of Science, Nanjing Forestry University, Nanjing 210037, China; njyp@njfu.edu.cn (P.Y.); wenzhounjfu@163.com (W.Z.); 15062225802@163.com (X.Z.); 2 Agricultural and Forest Products Processing Academician Workstation, Luohe 462600, China; njcjszx2005@163.com; 3 Post-Doctoral Research Center of Nanjiecun Group, Luohe 462600, China

**Keywords:** dialdehyde lignocellulose, thermoplastic starch, oxidation, content

## Abstract

In order to improve the mechanical properties and water resistance of thermoplastic starch (TPS), a novel reinforcement of dialdehyde lignocellulose (DLC) was prepared via the oxidation of lignocellulose (LC) using sodium periodate. Then, the DLC-reinforced TPS composites were prepared by an extrusion and injection process using glycerol as a plasticizer. The DLC and LC were characterized by X-ray diffraction (XRD) and scanning electron microscopy (SEM), and the effects of DLC content on the properties of the DLC/TPS composites were investigated via the evaluation of SEM images, mechanical properties, thermal stability, and contact angles. XRD showed that the crystallinity of the DLC decreased due to oxidation damage to the LC. SEM showed good dispersion of the DLC in the continuous TPS phase at low amounts of DLC, which related to good mechanical properties. The tensile strength of the DLC/TPS composite reached a maximum at a DLC content of 3 wt.%, while the elongation at break of the DLC/TPS composites increased with increasing DLC content. The DLC/TPS composites had better thermal stability than the neat TPS. As the DLC content increased, the water resistance first increased, then decreased. The highest tensile strength and elongation at break reached 5.26 MPa and 111.25%, respectively, and the highest contact angle was about 90.7°.

## 1. Introduction

Currently, research on biodegradable plastics is a very attractive field because of the increasing costs of synthetic plastics made from fossil sources and the environmental pollution caused by synthetic plastics [1]. Much attention has been paid to thermoplastic starch (TPS) as a renewable bioplastic material because of its abundance and low cost, and it is considered to be the most promising biodegradable plastic [2]. However, compared with synthetic plastics, TPS has disadvantages, including poor mechanical and thermal properties and pronounced sensitivity to moisture [3], which limits the possible applications of starch plastic products.

The properties of TPS can be improved by blending it with reinforcements such as organic fiber or inorganic mineral materials. Natural fibers, such as cotton fiber [4], flax fiber [5], coir fiber [6], luffa fiber [1], cassava bagasse cellulose [7], and wheat straw fiber [8], and inorganic mineral materials, including montmorillonite [9], kaolinite [10], halloysite [11], and rectorite [12], have been used to prepare TPS reinforcement composites. These reinforcements can improve the tensile strength, thermal stability, and water resistance seen in unblended TPS.

The amount of fiber added is a major factor affecting the properties of the TPS. Ma investigated the effects of different fiber contents of micro-winceyette on the mechanical properties of TPS [13]. They found that, with the increase in fiber content from 0 to 20 wt.%, the tensile strength increased 3 times, reaching 15.16 MPa; however, the elongation at break decreased from 105% to 19% which, according to the study by Prachayawarakorn [4], may have been due to the high crystallinity of the cellulose fiber. In Prachayawarakorn’s previous study [3], when the fiber content was 10 wt.%, the tensile strength reached a maximum and, moreover, the thermal stability at this fiber content was improved due to the higher and longer thermal resistance of the cellulose fibers [14]. A glycerol-plasticized pea starch/microcrystalline cellulose (MCC) composite showed good mechanical properties because of good dispersion [15]. At the same time, a study by Zhou showed that nanocellulose modified by oxidation could be dispersed homogeneously into polyvinyl alcohol (PVA) matrices [16], resulting in a superior tensile modulus and higher strength. However, in all of these studies, no fiber reacted with the TPS, which limited its ability to improve water resistance and mechanical properties.

Cellulose is the most abundant biopolymer on earth and is present in a wide variety of living species, such as animals [17], plants [18], and bacteria [19]. Dialdehyde cellulose has received a great deal of attention for over 40 years because of the high activity of aldehyde, which could be further used in many areas [20,21,22]. Moreover, thermoplastic dialdehyde starch (TPDAS) was reported to decrease biodegradation rates due to inter- and intramolecular cross-linking of dialdehyde starch in Du’s study [23]. At the same time, Yu found that the tensile strength of TPDAS increased with the increasing aldehyde content of dialdehyde starch contributed by cross-linking reactions [24,25]. Inspired by the abovementioned works, we proposed to combine the cross-linkable dialdehyde group with reinforced cellulose fiber into one system, that being dialdehyde cellulose, so the dialdehyde cellulose could react with the TPS effectively in the composite, and the corresponding mechanical properties and water resistance also could be improved.

In this study, dialdehyde lignocellulose (DLC) was oxidized from lignocellulose (LC) and used as reinforcement to prepare DLC/TPS composites. The objective was to have the DLC play the role not only of reinforcement, but also of a crosslinker, similar to dialdehyde starch, that could react with the starch in the traditional extrusion and injection process to improve the water resistance and mechanical properties of the TPS effectively. The effects of DLC content on the fracture morphology, mechanical properties, thermal properties, and surface water resistance of DLC/TPS composites were investigated in detail.

## 2. Results and Discussion

### 2.1. XRD Spectra

**Figure 1:**
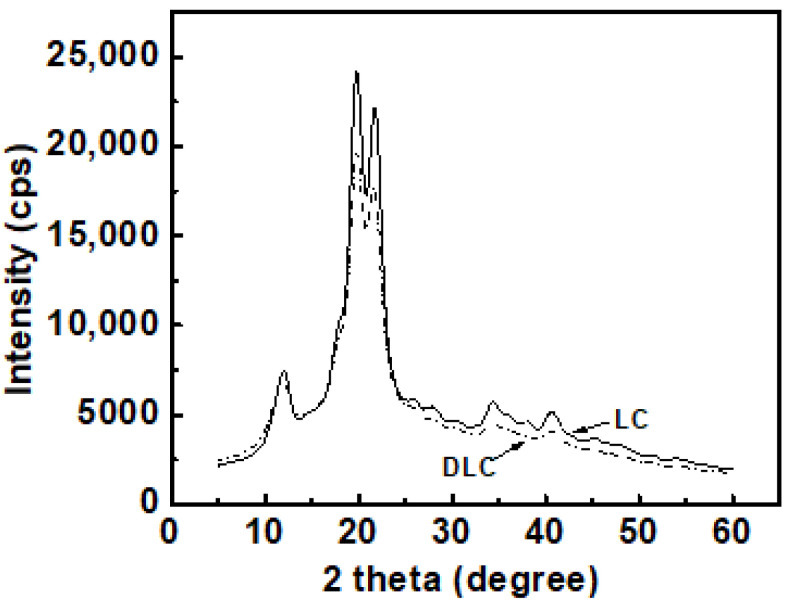
XRD spectra of lignocellulose (LC) and DLC.

Cellulose can be divided into crystalline and non-crystalline regions. The diffraction patterns of LC and DLC are presented in Figure 1. LC clearly possesses a characteristic crystalline region: the corresponding diffraction peaks occurred at 15.5° and 22.7°, which can be attributed to celluloseIIcrystallinity [26]. When the LC was oxidized to DLC, the positions of the diffraction peaks displayed no obvious changes. This implied that the main damage from the oxidation occurred in the amorphous region of the LC and that the crystalline structure had not been changed [26]; only the degree of crystallinity decreased slightly [27].

### 2.2. FTIR Spectra

**Figure 2:**
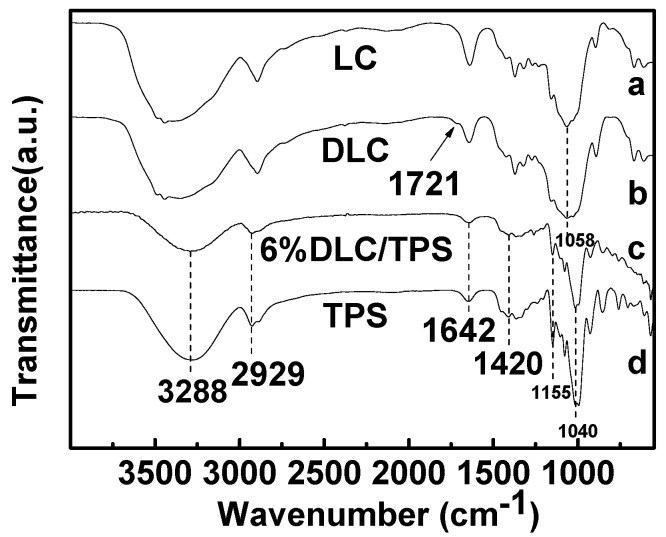
FTIR spectra of LC, DLC, TPS, and DLC/TPS composites.

Figure 2 shows the FTIR spectra of the LC, the DLC, the TPS, and the DLC/TPS composites (6%). In curves (a) and (b), the DLC showed a small shoulder peak at 1721 cm^−1^ compared with the LC after oxidation, which can be assigned to the C=O stretching vibrations of the aldehyde group and a weakened –OH absorbance at 3410 cm^−1^ and 1058 cm^−1^ [28,29]. This suggests that the aldehyde groups were introduced into the anhydroglucose unit (AGU) by the selective oxidation of periodate. It also indicates that the characteristic peak intensity of DLC is weak, which is due to the hemiacetal linkage between the resultant aldehydes (CHO) and hydroxyl groups not involved in the oxidation reaction of the AGU [28,29].

The FTIR spectrum of the TPS is shown in curve (d). The broad peak at 3288 cm^−1^ was dominated by the stretching of the OH groups. The peaks at 2929 cm^−1^ relate to C–H stretching. The peak at 1642 cm^−1^ was attributed to the bending mode of the absorbed water. The peaks around 1420 cm^−1^ are assigned to O–H bonding. The peaks at 1155 cm^−1^ and 1040 cm^−1^ were attributed to the C–O bond stretching of the C–O–H group in starch and the C–O bond stretching of the C–O–C group in the anhydroglucose ring, respectively [30,31]. In curve (c), the absorption bands at 1721 cm^−1^ disappeared, the –OH absorbance at 3288 cm^−1^ and 1420 cm^−1^ weakened, and the peaks at 1155 cm^−1^ and 1040 cm^−1^ also weakened, which could be due to the reaction between the –OH of the starch and the –CHO of the DLC in the DLC/TPS composites.

### 2.3. Mechanical Properties

**Figure 3:**
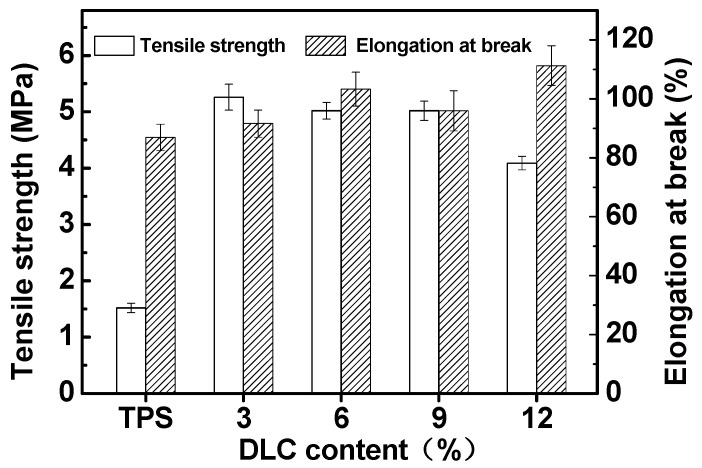
Effects of DLC content on the tensile strength and elongation at break of DLC/TPS composites.

Figure 3 shows the effects of DLC content on the mechanical properties of DLC/TPS composites. It can be seen that without DLC reinforcement, the TPS had poor tensile strength and elongation at break.

When the DLC content was increased from 0 to 3 wt.%, the tensile strength increased obviously from 1.52 to 5.26 MPa and the elongation at break increased a little, from 89.61 to 91.60%, due to the interfacial interaction between the DLC and the TPS matrix. When the DLC content was increased from 3 to 12 wt.%, the tensile strength decreased a little from 5.26 to 4.09 MPa, while the elongation at break increased from 91.60 to 111.25%. This phenomenon could be related to the emergence of phase separation and stress concentration that resulted from accumulated DLC with the higher DLC content. In addition, under these processing conditions, the presence of sugars produced during the oxidation processing of DLC was also reported to be a possible cause for the increase in elongation at break [32], and a similar unconventional increase the elongation at break with the addition of DLC was also reported in some studies [7,33]. Moreover, sugars also played the role of a plasticizer, which could decrease the tensile strength. Therefore, the DLC content of 3 wt.% produced the best reinforcement effect, with a tensile strength of 5.26 MPa and an elongation at break of 91.60%. This result could be due to the optimal content of DLC (3 wt.%) with the aldehyde content of 2.387%, which led to strong interfacial interactions between the DLC and the TPS matrix [24].

### 2.4. SEM

**Figure 4:**
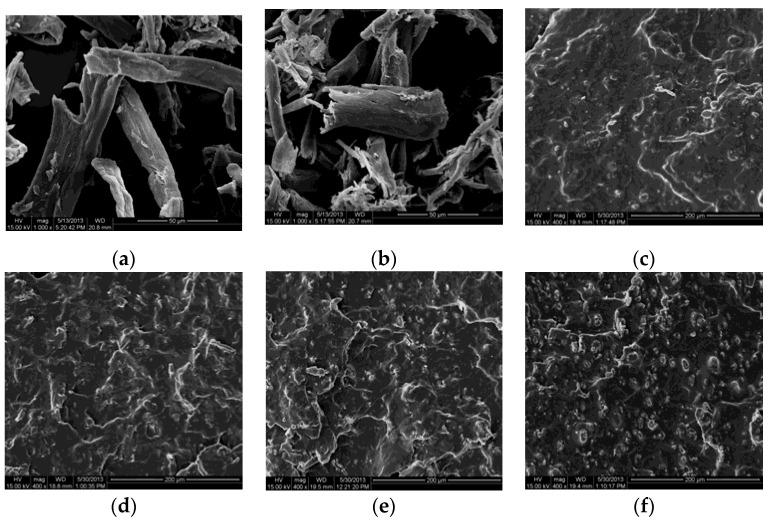
SEM micrographs showing the fracture surface of DLC/TPS composites (400×) with different DLC contents: (**a**) LC, (**b**) DLC, (**c**) 3 wt.% DLC/TPS, (**d**) 6 wt.% DLC/TPS, (**e**) 9 wt.% DLC/TPS, and (**f**) 12 wt.% DLC/TPS.

The morphologic structure of the composites is a very important characteristic because it ultimately determines many properties of the polymer composites. The SEM micrographs of LC and DLC, exhibiting the fractured surface of the DLC/TPS composites and revealing the dispersion of DLC within the TPS matrix, are shown in Figure 4a–f. It can be seen that the DLC (b) had a smaller size and a rougher fiber surface than the LC (a), which is due to the damage done by oxidation to the fibers.

As seen in Figure 4c–f, there was no starch granule structure present in the continuous DLC/TPS phase due to the high shear and temperature conditions in combination with the action of glycerol, and native corn starch granules melted or were physically broken up into small fragments [15]. The fractured surfaces of the DLC/TPS composites with low DLC contents were smooth, as seen in Figure 4c,d, and no obvious DLCs were observed on the surface, which contributed to the good mechanical properties. Moreover, the surface of the DLC was wetted and encapsulated by the TPS matrix, indicating better phase compatibility between the DLC and TPS due to their chemical cross-linking reactions. When the DLC content reached 9 wt.%, as seen in Figure 4e, some shiny spots from the DLC emerged due to some phase separation in the composite, which resulted in a decrease in the tensile strength of the DLC/TPS composites. Obvious shiny spots were observed from the DLC’s agglomeration in the DLC/TPS composites, especially for the 12 wt.% DLC content in Figure 4f.

### 2.5. Contact Angle Measurement

**Figure 5:**
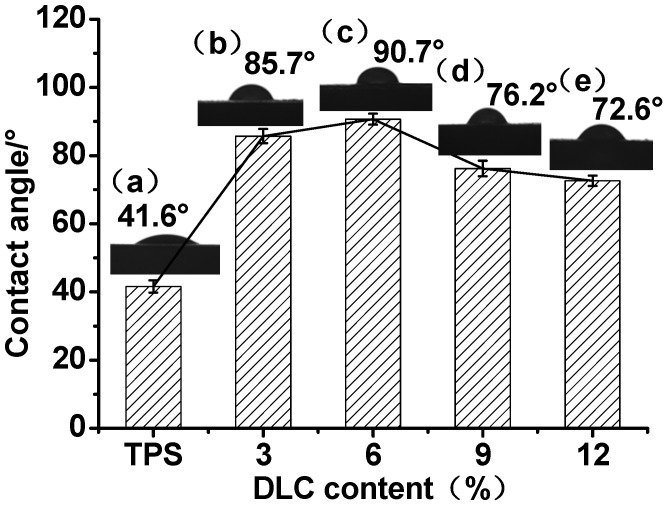
Contact angles of TPS and DLC/TPS composites: (**a**) TPS, (**b**) 3 wt.% DLC/TPS, (**c**) 6 wt.% DLC/TPS, (**d**) 9 wt.% DLC/TPS, and (**e**) 12 wt.% DLC/TPS.

Figure 5 shows the contact angles of the TPS and DLC/TPS composites. It can be seen that the contact angle for the TPS was 41.6°, and that with the addition of DLC, the contact angles became larger, as visualized in Figure 5b,c, indicating that the water resistance, via the cross-linking reaction between the aldehyde group in the DLC and the hydroxyl group in the TPS [24], was improved by the addition of the DLC. However, when the DLC content was 9 wt.%, the contact angle became smaller, and decreased even further at 12 wt.%. This phenomenon can be explained as follows: when the DLC content increased, more hydroxyl groups in the TPS reacted and were consumed, such that the original glycerol became relatively redundant, and this resulted in the increased hydrophilicity of the DLC/TPS composites. The DLC content of 6 wt.% produced the best water resistance, at a contact angle of 90.7°.

### 2.6. Thermal Stability

**Figure 6:**
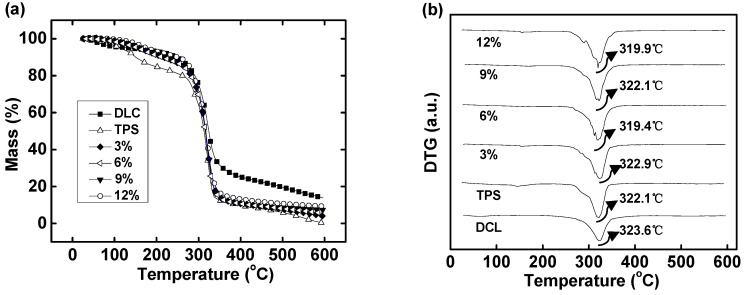
Thermogravimetric analysis of DLC, TPS and DLC/TPS composites: (**a**) TG curves; (**b**) DTG curves.

The TG and DTG curves of DLC/TPS composites under a nitrogen atmosphere are shown in Figure 6. From about 50 to 300 °C, the differences in mass loss between the DLC/TPS composite and the TPS indicated that the DLC/TPS composites had better thermal stability than the TPS. Below 150 °C, all weight losses were related to the volatilization of water or glycerol [13], which meant that the DLC/TPS composites contained less water and less free glycerol than did the TPS. This was due to the interaction between DLC and TPS, which reduced the water absorption. Moreover, DLC was able to react with the excess glycerol in the TPS.

In addition, as the DLC content increased, the thermal stability of the DLC/TPS composites became better; this may be due to a stronger interaction between DLC and TPS. However, as seen in the DTG curves in Figure 6, the peak temperature for the composite (that is, the temperature corresponding to the maximum decomposition rate for DLC/TPS composites with different DLC contents) was nearly the same as, although a little more than, that of the TPS, possibly due to their similar decomposition behavior under higher temperatures resulting from the similarities in chemical structure.

## 3. Materials and Methods

### 3.1. Materials

Corn starch (CS) was supplied by the Shandong Hengren Industry and Trade Company (Tengzhou, Shandong, China). The LC was homemade and based on bleached softwood pulpboard (a commercial product) made from oak bark. The glycerol, glycol, methanol, and sodium hydroxide were from Sinopharm Chemical Reagent Co., Ltd. (Shanghai, China). The sodium periodate was from Shantou Xilong Chemical Plant Co., Ltd. (Shantou, Guangdong, China). The chloride hydroxylamine was purchased from Shanghai No. 4 Reagent & H. V. Chemical Co., Ltd. (Shanghai, China). The sulfuric acid was from Nanjing Chemical Reagent Factory (Nanjing, Jiangsu, China). All chemicals were of analytical grade or similar.

### 3.2. Preparation of DLC

A piece of softwood pulpboard (50 g) was cut into small pieces and put into a 500 mL beaker. Then, 300 mL of a NaOH solution (15 wt.%) was added to the beaker to immerse the pulpboard. In fact, this is a process of swelling and activation for the pulpboard. At the same time, some impurities in the pulpboard were also removed in this process. After 5 h, the pH was about 10. Then, HCl (1.0 mol/L) was added to neutralize the solution to pH 7. Finally, the deposit was filtered using a sand core funnel and washed with distilled water. The deposit was dried at 50 °C for 8 h.

The sample and 60 wt.% H_2_SO_4_ were added to a 500 mL beaker for the hydrolysis reaction at 45 °C for 0.5 h under constant stirring (200 rpm). Then, the slurry was centrifuged with distilled water repeatedly at 3000 rpm to remove acids and other small molecules. The deposit was dried by a freeze dryer, and the LC was obtained.

Next, 250 mL of deionized water and sodium periodate (5 g) were added to a 500 mL flask in a water bath held at constant temperature (50 °C). The pH of the solution was about 5, then adjusted to pH 4.0 using a small amount of HCl (1.0 mol/L) solution. Then, 20 g of LC was added rapidly to the flask under lightproof conditions for a reaction lasting 3 h with constant stirring (200 rpm). Finally, ethylene glycol (50 mL) was added to remove the unreacted sodium periodate completely for about 1 h, and DLC was obtained after filtering, washing, and drying.

### 3.3. Determination of Aldehyde Group Content

The aldehyde contents of DLC were determined according to a study by Yu [24], with some modifications. DLC (0.5 g) was added to the hydroxylamine hydrochloride (10 mL of 0.05 g/mL) solution. The pH was adjusted to 5 using a NaOH (0.1 mol/L) solution. The conversion of aldehydes into oximes continued at 40 °C for 4 h. The aldehyde content was determined according to Equation (1) by recording the consumption V_1_ (mL) of NaOH (0.1 mol/L) and performing the reaction at a constant pH of 5. To record the consumption V_2_ (mL) of NaOH, 0.5 g of LC without oxidation was used as a control.

Aldehyde content is calculated as:

(1) $$CHO(\%)=\frac{M(CHO)\times c\times (V_{1}-V_{2})}{m\times 1000}\times 100$$

where *M (CHO)* is the molar mass of the –CHO group (29 g/mol); *c* is the concentration of NaOH (0.1 mol/L); *V*_1_ is the volume of the consumption of NaOH for DLC (mL); *V*_2_ is the volume of the consumption of NaOH for LC without oxidation (mL); and *m* is the mass of DLC or LC (0.5 g).

The aldehyde content of the DLC was calculated to be 2.378%.

### 3.4. Preparation of DLC/TPS Composites

**Figure 7:**
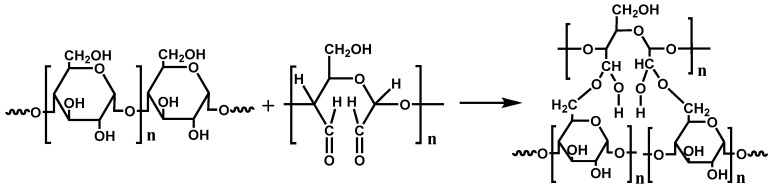
Reaction equation of cross-linking reaction between dialdehyde lignocellulose (DLC) and thermoplastic starch (TPS).

**Figure 8:**
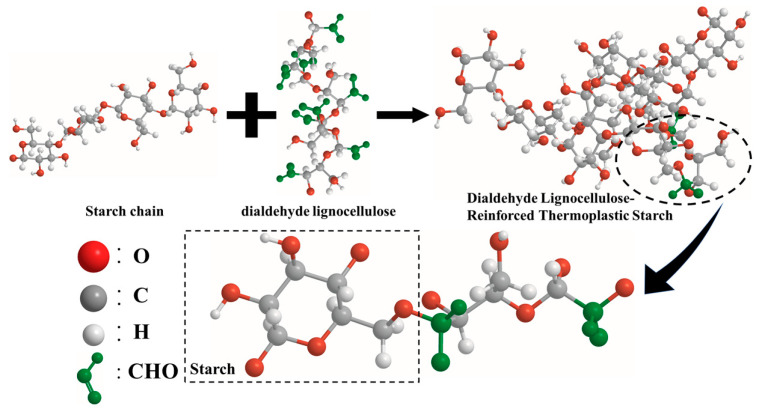
Schematic illustration of the potential chemical interactions between DLC and TPS.

Glycerol was blended with CS and DLC using a high-speed mixer (GH-100Y, made in Beijing, China) and then stored overnight. The ratio of glycerol to CS (*w*/*w*) was 1:3 [34]. The DLC loading level (0, 3, 6, 9, or 12 wt.%) was based on the mass sum of CS and glycerol. The mixtures were transferred into a twin-screw extruder (SHJ-20, Nanjing Giant Machinery Co., Ltd., Nanjing, China) with a screw speed of 180 rpm, and the four zone temperatures were 115, 120, 125, and 115 °C, respectively. The length of the barrel was 80 mm, the die had a hole of 3 mm in diameter, and the throughput of the material at the set conditions was 0.6 kg/h. After extrusion, samples were cooled and cut into small particles by a Pelletizer. Next, an injection molding machine (BV90, APOLLO, Shanghai, China) was used to inject a dumbbell-shaped specimen, and the temperatures in each zone were 135, 135, 125, 125, and 120 °C, with a pressure of 60 MPa, according to our previous study [34]. Each sample was prepared for five tested specimens at least. The reaction equation and schematic illustration between DLC and TPS are shown in Figure 7 and Figure 8, respectively.

### 3.5. X-Ray Diffraction (XRD) Analysis

The injected dumbbell-shaped specimen was cut into samples with a thickness of approximately 2 mm, a width of 10 mm, and a length of approximately 10 mm, and was applied to the surface of the glass mold tank with double-sided tape. Then, the prepared samples were directly irradiated. The X-ray diffraction patterns of the samples were recorded in the reflection mode at ambient temperature by an X-ray diffractometer (Ultima IV., Rikagu, Japan). The ray source was Cu Kα, the scanning angle range was 5°–60°, the angle step was 0.02°, and the test tube voltage current was 30 kV and 30 mA.

### 3.6. Fourier Transform Infrared Spectroscopy (FTIR) Analysis

The absorbance spectra of the LC, the DLC, the TPS, and the DLC/TPS composites were recorded with an infrared spectrometer (VERTEX 70, Bruker, Germany) in attenuated total reflectance (ATR) mode to investigate the variation in functional groups. The spectra were obtained in the range of 4000–400 cm^−1^, and the resolution of the FTIR was 2 cm^−1^.

### 3.7. Mechanical Tests

Measurements of mechanical properties were performed in accordance with the ASTM D638 standard. The tensile measurements were conducted on a testing machine (SANS, Shenzhen, China). Dumbbell-shaped specimens (2 mm thick) of the composites were obtained by injection molding. The samples were placed between the grips of the machine and stretched with a strain rate of 20 mm/min at room temperature. Five to eight specimens were tested for each sample, and the average values and SD of the measured properties were reported.

### 3.8. Scanning Electron Microscopy (SEM) Analysis

The LC and DLC powders and the fracture surfaces of the DLC/TPS composites were examined using a field emission scanning electron microscope JEOL JSM-7600F (Takeno, Tokyo, Japan). The LC and DLC powders were dispersed into ethanol and then water for 5 min each by way of ultrasonication. The suspension drops were drawn on a glass flake, dried to remove the ethanol or water, and then vacuum coated with gold for SEM imaging. The DLC/TPS composites were broken in the tensile tests. The fracture faces were vacuum coated with gold for SEM imaging.

### 3.9. Contact Angle Measurement

The contact angle of the DLC/TPS composites was measured by contact angle testing (DSA100, Kruss, Hamburg, Germany). It should be noted that the contact angle testing results of the samples were obtained by averaging 10 independently tested specimens, and that the contact angle was tested 20 s after the water droplets had fallen into the specimen.

### 3.10. Thermal Stability Studies

Thermogravimetric (TG) and differential thermogravimetric (DTG) curves of the samples were recorded on a thermogravimetric analyzer (TG 209 F1, Netzsch, SELB, Bavaria, Germany), at a heating rate of 20 °C/min. The samples were heated from ambient temperature to 600 °C to determine the complete thermal degradation of the DLC and the DLC/TPS composites. All tests were carried out in a nitrogen atmosphere with a 20 mL/min flow rate.

## 4. Conclusions

In the present study, it can be seen from the XRD and FTIR spectra that LC was oxidized to DLC successfully. SEM showed good dispersion of DLC in the continuous TPS phase, but some phase separation appeared at higher DLC contents. DLC addition improved the mechanical properties of the material via the strong interfacial interaction related to the cross-linking reaction. The tensile strength of DLC/TPS reached its maximum (5.26 MPa) at the DLC content of 3 wt.%, the elongation at break of the DLC/TPS composites increased with increasing DLC content, and the highest elongation at break reached 111.25%. The DLC/TPS composites exhibited better thermal stability than did the TPS, and as the DLC content increased, the thermal stability of DLC/TPS composites became better. With the increase in DLC content, the contact angle of the DLC/TPS composites first increased, then decreased. The best water resistance, with a contact angle of 90.7°, occurred when the DLC content was 6 wt.%.
